# A three-year longitudinal study of retinal function and structure in patients with multiple sclerosis

**DOI:** 10.1007/s10633-021-09855-7

**Published:** 2021-10-27

**Authors:** James V. M. Hanson, Mei-Yee Ng, Helen K. Hayward-Koennecke, Sven Schippling, Kelly A. Reeve, Christina Gerth-Kahlert

**Affiliations:** 1 Department of Ophthalmology, University Hospital Zurich and University of Zurich, Frauenklinikstrasse 24, 8091 Zurich, Switzerland; 2 Masters Program in Biostatistics, University of Zurich, Hirschengraben 84, 8001 Zurich, Switzerland; 3 Clinic for Neurology, Neuroimmunology and Multiple Sclerosis Research, University Hospital Zurich and University of Zurich, Frauenklinikstrasse 26, 8091 Zurich, Switzerland; 4 Multimodal Imaging in Neuroimmunological Diseases (MINDS), University Hospital Zurich and University of Zurich, Frauenklinikstrasse 26, 8091 Zurich, Switzerland; 5 Institute for Epidemiology, Biostatistics, and Prevention, Department of Biostatistics, University of Zurich, Hirschengraben 84, 8001 Zurich, Switzerland

**Keywords:** Multiple sclerosis, Electroretinogram, Optical coherence tomography, Retina, Bipolar cells

## Abstract

**Background:**

Researchers have in recent years begun to investigate ophthalmological manifestations of multiple sclerosis (MS) other than optic neuritis (ON), and it is now clear that changes to retinal function (measured using the electroretinogram, ERG) and structure (measured using optical coherence tomography, OCT) are found in MS patients irrespective of prior ON episodes. ERG results are consistent with dysfunctional bipolar cells, as in other autoimmune diseases. To date, studies have presented only cross-sectional data regarding ERG and OCT. We, therefore, studied the longitudinal course of ERG and OCT in patients with MS, as well as the effect of disability changes and non-ON clinical relapses on these functional and structural measures.

**Methods:**

MS patients (*n* = 23) participating in an ongoing longitudinal observational study were invited to take part in a 3-year ophthalmological substudy. ERG and OCT were performed, and measures of MS-related disability and relapse history were obtained. Study visits were repeated annually. ERG peak times, rod b-wave amplitude, mixed rod/cone and cone b-/a-wave amplitude ratios, thickness of the peripapillary retinal nerve fibre layer, and volumes of the segmented retinal layers/complexes were analysed. Using generalised estimating equation models adjusted for age, ON, and MS treatment status, we assessed changes to ERG and OCT over the study duration, the effect of changes in disability and recent non-ON MS relapses on ERG and OCT, and the effect of selected OCT parameters on corresponding ERG parameters.

**Results:**

At the group level, small fluctuations of several ERG peak times were recorded, with OCT values remaining stable. Increased disability between visits was associated with significant prolongation of mixed rod-cone ERG b-wave peak times. No evidence of associations between OCT and ERG parameters was observed.

**Conclusions:**

Retinal bipolar cell function may be affected by changes in disability in patients with MS; however, recent non-ON MS clinical relapses appear not to affect ERG or OCT results. As ERG changes in MS patients over 3 years are likely to be small and of uncertain clinical relevance, longitudinal studies of retinal function in MS should be planned over an extended period.

**Supplementary Information:**

The online version contains supplementary material available at 10.1007/s10633-021-09855-7.

## Introduction

Multiple sclerosis (MS) is a chronic neurological autoimmune disease characterised by inflammatory demyelination and neurodegeneration within the central nervous system (CNS). In approximately 90% of cases, MS manifests with a first clinical episode suggestive of demyelination in the CNS without the evidence of dissemination of disease activity in time and space required for a diagnosis of MS [1]; such episodes are referred to as clinically isolated syndrome (CIS) [1]. Despite a heterogeneous clinical presentation, the afferent visual pathway is frequently affected in patients with MS and CIS, with optic neuritis (ON) being particularly common [2]. The high prevalence of visual system involvement, together with the accessibility of the retina as the only location where unmyelinated axons and neurons can be examined in vivo in humans, has generated increasing interest in the afferent visual pathway as a model for MS [3, 4]. Retinal function and structure can be measured with the full-field (ganzfeld) electroretinogram (ERG) and with optical coherence tomography (OCT), respectively. Photoreceptor function is captured predominantly by the ERG a-wave, with the ERG b-wave and flicker response reflecting predominantly bipolar (post-receptoral, pre-ganglionic) function [5, 6]. Structural examination has been facilitated by the development of software tools enabling the demarcation and quantitative analysis of the retinal layers (‘segmentation’) from OCT scans.

In recent years, the ERG has enabled the documentation of outer retinal dysfunction in patients with MS, particularly affecting bipolar cells driven wholly or partly by the cone photoreceptors [7–12]. This dysfunction, evidenced by prolongation of the ERG b-wave and/or 30 Hz flicker response peak time, appears independent of a history of ON [7, 11] and has been demonstrated in cohorts where eyes with ON were excluded [8–10]. Bipolar dysfunction has also been documented in other autoimmune diseases [13–21]. Delayed ERG a-wave peak times have also been documented [7, 9–12], although not by all investigators [8, 22]. Using OCT in addition to ERG, we have demonstrated that the abnormal ERG-derived measures of bipolar cell function in patients with MS are uncorrelated with structural measures of their presumed retinal origin, the inner nuclear layer (INL) [7], consistent with dysfunctional, but not atrophic, bipolar cells [7, 11]. Despite these robust cross-sectional findings, documented by a number of groups working independently, longitudinal functional data regarding the bipolar cells and INL in patients with MS are currently sparse.

Longitudinal OCT-derived retinal structural studies in patients with MS have recorded accelerated thinning of the retinal nerve fibre layer (RNFL) and ganglion cell-inner plexiform layer (GCIPL) relative to control subjects [23–26]. Whilst ON leads to variable atrophy of RNFL and GCIPL, this transient insult does not significantly affect the underlying rate of insidious inner retinal thinning [24, 25]. A recent multi-centre study focussing on INL documented thickening (as opposed to thinning) after ON or non-ON clinical relapses [27], compatible with earlier findings describing increased INL thickness in patients with active MS [28]. Conversely, some authors have reported mild longitudinal INL thinning in patients with MS [26]. In summary, INL findings in MS appear to be more variable than ERG results and, importantly, confounded by ON. Conversely, ERG findings are broadly consistent between different research groups and appear to be unaffected by ON (as discussed above). The extent to which ERG findings reflect clinical changes in MS status remains unknown.

With these factors in mind, we set out to longitudinally measure ERG and OCT in patients with MS and CIS, determine the relationships between these functional and structural outcome measures, and investigate the influence of clinical relapses and changes in global disability on retinal function and structure.

## Methods

All subjects were participants in an ongoing longitudinal study of MS at the University Hospital Zurich who consented in writing to participate in a three-year longitudinal ophthalmological substudy. Inclusion criteria for the substudy were: confirmed diagnosis of MS or CIS according to contemporary criteria [29], and age at enrolment 18–65 years. Exclusion criteria were: refractive errors > 6 dioptres, co-existing ocular or neurological disease other than MS, and diabetes mellitus. The study adhered to the tenets of the Declaration of Helsinki and was approved by the Cantonal Ethics Committee of Zurich (EC-No.2013–0001). Examinations consisted of: best-corrected high- and low-contrast visual acuity using Early Treatment Diabetic Retinopathy Study (ETDRS) and 2.5% contrast Sloan charts, respectively; anterior segment and mydriatic fundus examination by a senior ophthalmologist, OCT, and ERG. Following baseline examinations, this battery of tests was repeated on an annual basis for three years, making a total of four examinations per subject. Examinations also included measurement of the visual evoked potentials, multifocal ERG, pattern ERG, and photopic negative response; however, the results of these examinations were not analysed longitudinally due to our focus on measures of panretinal bipolar function. Expanded Disability Status Scale (EDSS) score and relapse history were obtained by neurological examination and chart review by experienced neurologists. As EDSS values were < 5.5 throughout the study for all patients, a change in EDSS of 1.0 or more between examinations was considered clinically meaningful [30]. All examinations took place over the period June 2014–October 2019.

### ERG

ERG was recorded using gold-plated skin electrodes and single-use DTL (Dawson et al.) recording electrodes (Diagnosys LLC, Lowell MA, U.S.A) according to contemporary standards of the International Society for Clinical Electrophysiology of Vision [31] on an Espion system (Diagnosys LLC), which was annually calibrated throughout the duration of the study. Medical mydriasis was accomplished using topical 0.5% tropicamide and 5% phenylephrine. Topical 0.4% oxybuprocaine was instilled prior to positioning the DTL electrodes. All ERG measurements were made by a single experienced operator (author JVMH) using identical electrodes and with the DTL thread positioned horizontally at the lower lid margin. Recordings were made with a bandwidth of 0.3–300 Hz and a sampling rate of 2 kHz.

After 20 min of dark adaptation, patients were presented with 0.01 cd/m^2^ flashes (‘rod’) followed by 3.0 cd/m^2^ flashes (‘rod-cone’). Following these measurements, patients were adapted to a rod-bleaching 30 cd/m^2^ light for 10 min before being presented with 3.0 cd/m^2^ light, both flickering (30 Hz frequency; ‘flicker’) and single flashes (‘cone’) against a 30 cd/m^2^ background. All stimuli were presented via a full-field stimulator with diffusor, of 4 ms duration, and composed of white light. Multiple responses were recorded for each condition to verify reproducibility, which were then averaged.

From the ERG, the a-wave, b-wave, and flicker peak times and amplitudes were ascertained for each eye and each stimulus condition with the exception of the dark-adapted (DA) 0.01 cd/m^2^ (‘rod’) a-wave, which is not recommended for quantitative analysis [31]. Ratios of the rod-cone and cone b-wave/a-wave amplitudes (a normalised measure of bipolar function) were calculated. All ISCEV standard [31] ERG peak times were included in our analysis. The rod b-wave amplitude was also analysed, due to previous work suggesting longitudinal changes to this parameter (and, to a lesser extent, other ERG amplitudes) in MS patients [10]. Other ISCEV standard ERG amplitudes were not analysed due to the predominance of normal findings in previous MS studies [7–12], and because the known dependence of amplitudes (but not peak times) on DTL position [32, 33] could have potentially confounded longitudinal measures of ERG amplitudes. Instead, we captured ERG amplitudes using the rod-cone and cone b-wave/a-wave ratios.

### OCT

All OCT scans were acquired in a darkened room using a Spectralis device (Heidelberg Engineering, Heidelberg, Germany. High resolution circumpapillary scans (12° diameter; 100 Automatic Real-time Tracking [ART]) were aligned to the visible centre of the optic nerve head, whilst high resolution volume scans (30° vertical by 15° horizontal,19 vertically oriented sections separated by 240 µm, 25 ART) were centred on the fovea. Baseline scans were set as the reference within the Heidelberg software, with scans at visits 2, 3, and 4 acquired in ‘follow-up’ mode to ensure precise alignment of subsequent scans with the same anatomical landmarks as at baseline. After ensuring that all acquired OCT scans were of acceptable quality as defined by the OSCAR-IB criteria [34], the volume scans were automatically segmented and manually verified and corrected using proprietary software (Heidelberg Engineering). This enabled visualisation and quantification of the macular ganglion cell-inner plexiform layer complex (GCIP), INL, outer plexiform layer (OPL), ONL, and outer retinal layers (ORL; defined proximally by the external limiting membrane [ELM] and distally by Bruch’s membrane [BM], and therefore comprising mainly the photoreceptors) for each eye. Each of the macular OCT parameters was summarised as the volume (in mm^3^) of each layer or complex measured over a 3.45 mm diameter circle. RNFL thickness measurements were obtained from the circumpapillary OCT scan; the global thickness (RNFL-G), averaged from all sectoral measurements, was analysed, along with thickness in the temporal (RNFL-T) and papillomacular bundle (RNFL-PMB) sectors. All OCT scans were acquired and verified by a single experienced operator (author JVMH).

### Statistical Analyses

Specific goals of the study were as follows:

- To ascertain whether ERG and OCT parameters changed over the 3-year study duration
- To investigate whether changes to MS clinical status (EDSS score; MS relapse within the previous 12 months) were associated with changes in ERG and OCT parameters
- To evaluate whether changes in OCT parameters (INL; ONL and ORL) were related to changes in the relevant ERG outcome measures (ERG b-wave and flicker peak times, ERG b/a wave ratios; ERG a-wave peak times) over the study duration

Both eyes of each patient were analysed. Generalised estimating equation (GEE) models [35] assuming independent correlation structure were used in order to account for both longitudinal correlations and correlations between measurements of different eyes of the same patients. All models were adjusted for age, previous ON, and MS treatment status (treated/untreated). ON history was determined for each eye separately, whereas age, EDSS score, MS relapse history, and MS treatment status were identical for both eyes. *P* values were corrected for multiple comparisons using the method of Benjamini and Hochberg [36]. Corrected *p* values < 0.05 were considered significant. All analyses were performed in R version 3.5.3 [37] using the library geepack version 1.3.1 [38]. Graphs were created initially in *R*, with Figs. 2 and 3 being assembled from individual graphs in Affinity Designer version 1.7.3.481 (Serif [Europe] Ltd, Nottingham, U.K.).

## Results

After dropouts, longitudinal data from 23 patients aged 23–54 years were available for analysis. 14 patients had relapsing–remitting MS (RRMS), eight CIS, and one primary progressive MS (PPMS). Median disease duration, defined as time since first MS/CIS symptoms, was 20 months. One eye of a single patient was excluded due to a large-angle exotropia, meaning that 45 eyes were analysed. (However, OCT of a single patient at baseline failed quality control and was excluded from analysis.) Reliable HCVA and LCVA values could not be obtained at baseline for both eyes of a single patient due to an accommodative spasm. It was not possible to quantify HCVA and/or LCVA at baseline in a small number of additional eyes (HCVA: *n* = 1; LCVA: *n* = 2) with previous ON, as the patients were unable to read any letters on the test charts. 16 eyes had previous ON, with three of these having ON within the 12 months prior to baseline; one patient experienced unilateral ON during the course of the study. Seven patients experienced non-ON clinical relapses over the study period. Of the eight patients with CIS at baseline, five had converted to MS by the end of the study. Demographic details of the patient cohort at baseline are given in Table 1.

**Table 1:** Characteristics of the patient cohort at baseline. CIS, clinically isolated syndrome; EDSS, Expanded Disability Status Scale; HCVA, high-contrast visual acuity; IQR, interquartile range; LCVA, low-contrast visual acuity; MAR, minimum angle of resolution; ON, optic neuritis; PPMS, primary progressive multiple sclerosis; RRMS, relapsing–remitting multiple sclerosis; SD, standard deviation. Disease duration is defined as the time since initial MS/CIS symptoms

	Level	Value
Patients (total eyes analysed) Diagnosis		23 (45)
	RRMS	14 (61%)
	CIS	8 (35%)
	PPMS	1 (4%)
Treatment	No	12 (52.2%)
	Yes	11 (47.8%)
Relapse (previous 12 months)	No	16 (69.6%)
	Yes	7 (30.4%)
ON	No	29 eyes (64.4%)
	Yes	16 eyes (35.6%)
ON (previous 12 months)	No	42 eyes (93.3%)
	Yes	3 eyes (6.7%)
Age (years) (median [IQR]; mean (SD))		38.00 [31.00, 43.50]; 37.13 (8.97)
Disease duration (months) (median [IQR]; mean (SD))		20.00 [12.00, 72.00]; 59.83 (82.16)
EDSS (median [minimum, maximum])		1.5 [0, 4]
HCVA (logMAR) (median [IQR]; mean (SD))		-0.09 [-0.13, -0.06]; -0.09 (0.07)
2.5% LCVA (logMAR) (median [IQR]; mean (SD))		0.42 [0.36, 0.57]; 0.46 (0.16)

Although the majority of patients attended all examinations, a minority were unable to attend the second and/or third examinations for reasons such as pregnancy/childbirth or a change in location. However, all 23 patients attended both the first and last examinations, permitting analysis of functional and structural retinal changes over a three-year period. OCT was not possible in one eye of a single patient at visit 3 only, due to severe visual loss after ON and consequent malfixation. The number of eyes analysed at each visit are recorded in Tables S1 (ERG) and S2 (OCT).

**Fig. 1:**
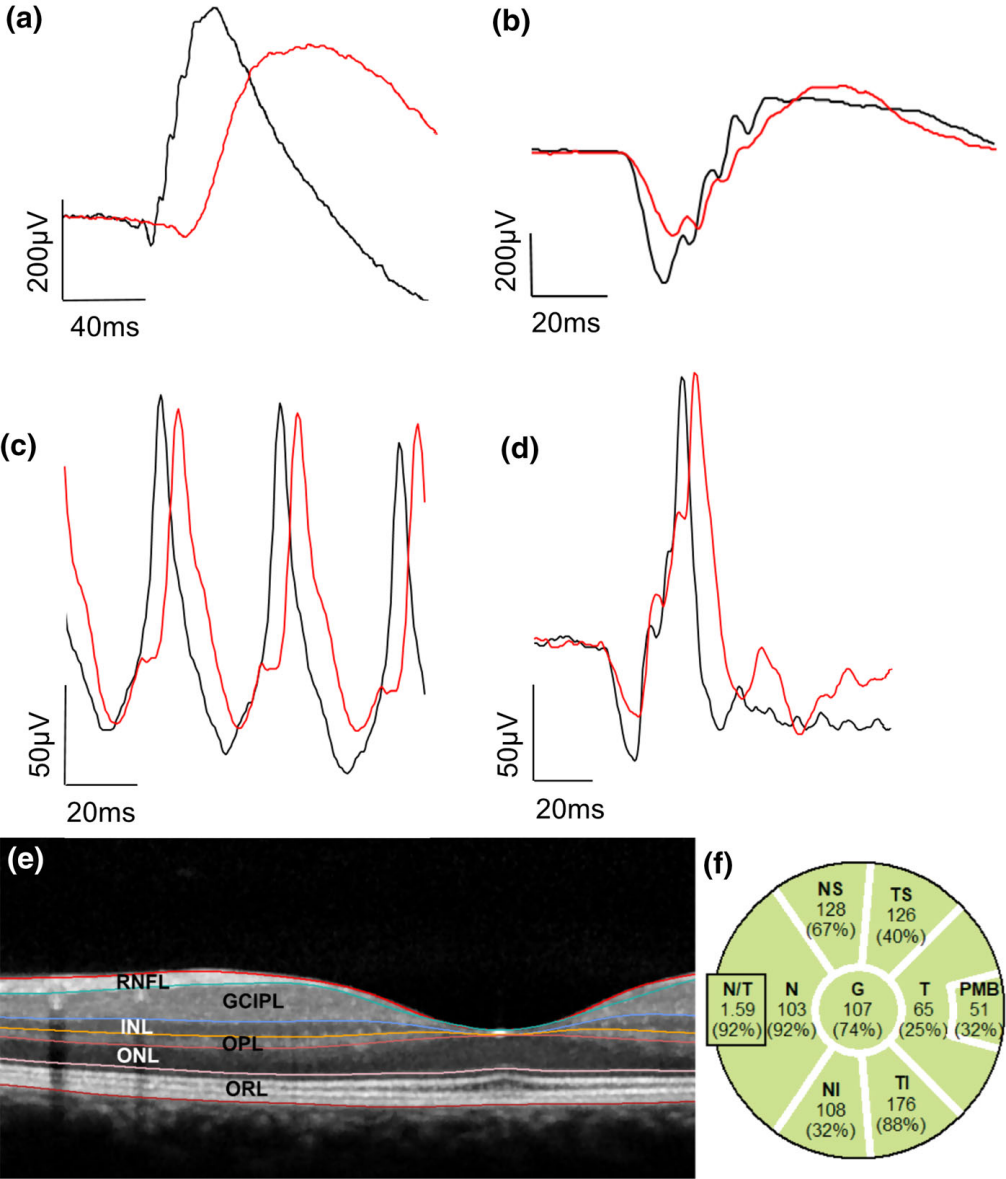
**a**–**f**. Representative ERG waveforms (**a**–**d**) and illustration of the OCT layers and complexes analysed. ERG waveforms are shown for a healthy individual (black curves) and a patient with MS (red curves) following dark-adapted 0.01 (‘rod’), dark-adapted 3.0 (‘rod-cone’), light-adapted 3.0 30 Hz (‘flicker’), and light-adapted 3.0 single flash (‘cone’) stimulation (a-d, respectively). For all conditions, x-axis scales represent time since stimulus onset in milliseconds (ms), and y-axis scales amplitudes in µV. Note that no normative patients were included in the present study; the waveforms are shown to illustrate cross-sectional findings in patients with MS, rather than the longitudinal course of ERG measurements described in this work. A segmented OCT macular scan with the relevant layers and complexes highlighted (e) and the circumpapillary thickness analysis of RNFL (f) are displayed. Only G, T, and PMB were included in the analyses. The values for each sector/quadrant show the thickness in µm, with the figure in brackets showing the corresponding percentile of age-matched normal values. G, global average of RNFL thickness; GCIPL, ganglion cell-inner plexiform layer; INL, inner nuclear layer; N, nasal quadrant of RNFL; NI, nasal inferior sector of RNFL; NS, nasal superior sector of RNFL; N/T, ratio of nasal/temporal RNFL thickness; ONL, outer nuclear layer; OPL, outer plexiform layer; ORL, outer retinal layers; PMB, papillomacular bundle; RNFL, retinal nerve fibre layer; S, superior quadrant of RNFL; T, temporal quadrant of RNFL; TI, temporal inferior sector of RNFL; TS, temporal superior sector of RNFL

**Fig. 2:**
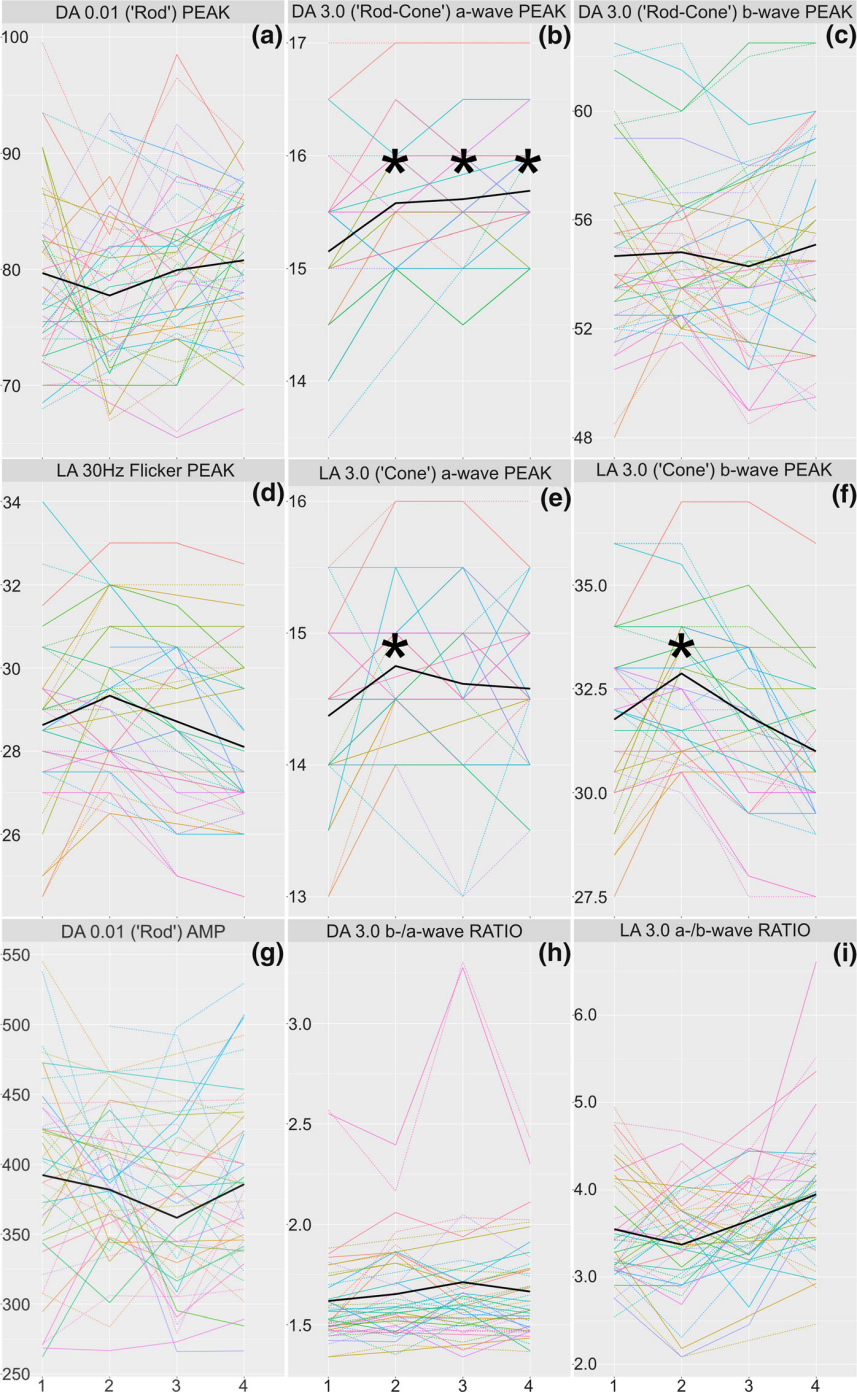
**a–i** Plot showing analysed ISCEV standard ERG parameters over the study duration, including all peak times (‘PEAK’; **a**–**f**), DA 0.01 amplitude (‘AMP’; g), and DA 3.0 and LA 3.0 b-/a-wave ratios (‘RATIO’; **h**–**i**). Coloured lines show the parameters of individual eyes (right eyes: dashed lines; left eyes: solid lines). Individual patients are represented with different colours. Solid black lines show results at the level of the entire cohort (see also Table 2). Results at individual visits which differ significantly from baseline (visit 1) are highlighted with black asterisks. DA, dark adapted; LA, light adapted

**Fig. 3:**
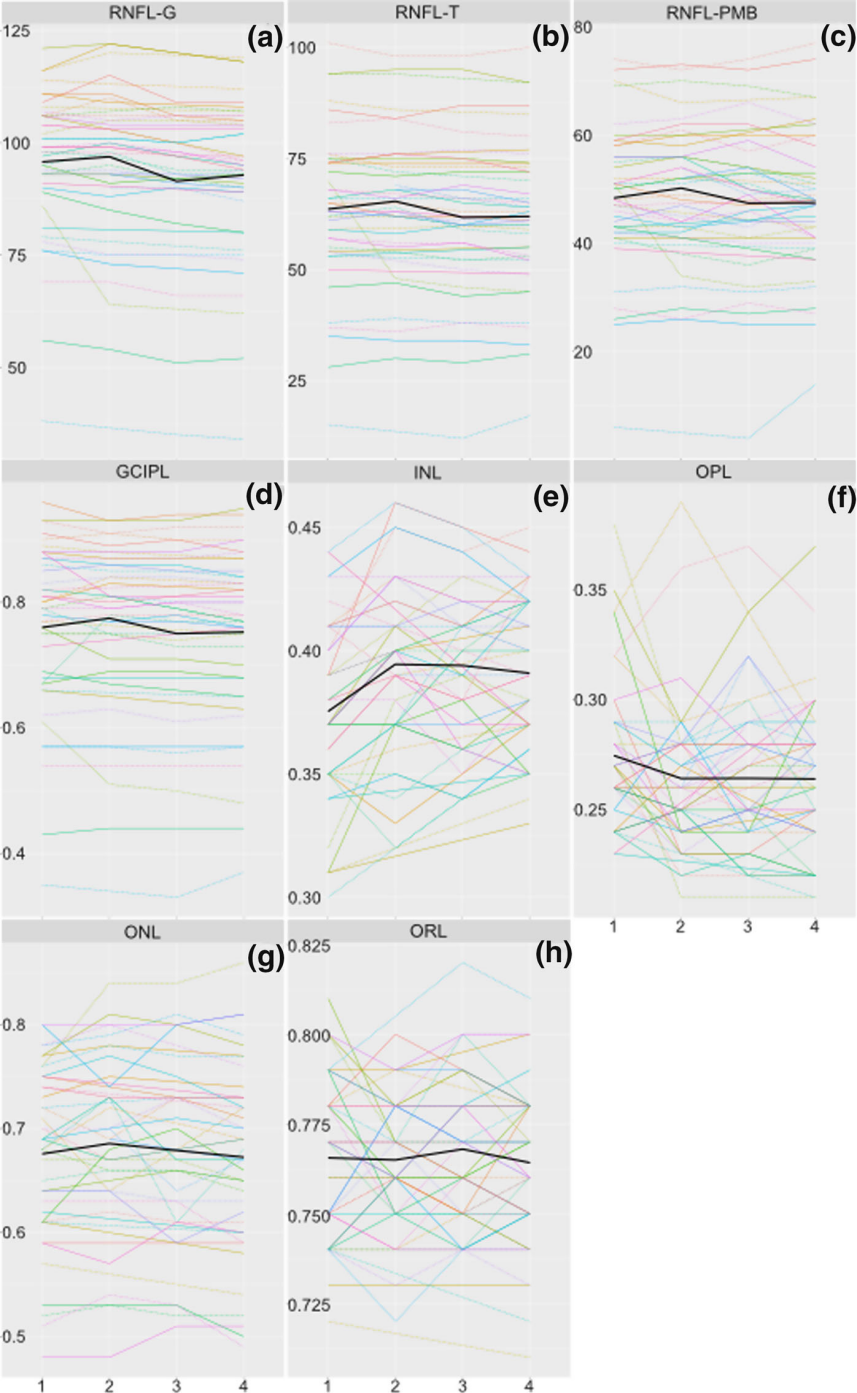
**a–h** Plot showing analysed OCT parameters over the study duration. Coloured lines show the parameters of individual eyes (right eyes: dashed lines; left eyes: solid lines). Individual patients are represented with different colours. Solid black lines show results at the level of the entire cohort (see also Table 3). All parameters at visits 2, 3, and 4 did not differ significantly from baseline (visit 1). G, global average of RNFL thickness; GCIPL, ganglion cell-inner plexiform layer; INL, inner nuclear layer; ONL, outer nuclear layer; OPL, outer plexiform layer; ORL, outer retinal layers; PMB, papillomacular bundle; RNFL, retinal nerve fibre layer; T, temporal quadrant of RNFL

Illustrative cross-sectional ERG waveforms from a participant with MS are shown, along with those of a healthy individual, in Fig. 1a–d. Mean and median ERG and OCT data at each visit are provided in Tables S1 and S2, respectively. The results of the GEE models analysing ERG and OCT findings over time are displayed in Table 2 and Fig. 2, and Table S3 and Fig. 3, respectively. At the group level, we recorded significant changes in the following ERG parameters (all relative to the visit 1 baseline): DA 3.0 a-wave peak time (visits 2, 3, and 4), LA 3.0 a-wave peak time (visit 2), and LA 3.0 b-wave peak time (visit 2). We did not observe any significant changes in other ERG parameters, or in any OCT parameters, over the study duration.

**Table 2:** Results of GEE models describing changes in ERG parameters over the study duration

ERG Variable	Covariate	Coefficient	95% CI	P
DA 0.01 ('Rod') **AMP**	Intercept	394.94	309.39 to 480.48	NA
	Visit 2	− 9.56	− 36.86 to 17.74	0.83
	Visit 3	− 29.85	− 57.3 to -2.41	0.14
	Visit 4	− 5.77	− 27.38 to 15.85	0.86
DA 0.01 ('Rod') **PEAK**	Intercept	72.15	63.48 to 80.81	NA
	Visit 2	− 2.30	− 6.29 to 1.7	0.50
	Visit 3	− 0.46	− 3.76 to 2.84	0.93
	Visit 4	0.43	− 3.05 to 3.91	0.93
DA 3.0 (Rod-Cone') a-wave **PEAK**	Intercept	13.96	13.13 to 14.79	NA
	Visit 2	0.36	0.1 to 0.62	**0.037**
	Visit 3	0.36	0.15 to 0.56	**0.005**
	Visit 4	0.43	0.25 to 0.61	**0.0001**
DA 3.0 ('Rod-Cone') b-wave **PEAK**	Intercept	56.11	50.69 to 61.53	NA
	Visit 2	0.20	− 0.76 to 1.17	0.92
	Visit 3	− 0.26	− 1.07 to 0.54	0.83
	Visit 4	0.54	− 0.5 to 1.58	0.55
DA 3.0 b/a-wave **RATIO**	Intercept	0.77	0.19 to 1.35	NA
	Visit 2	0.00	− 0.08 to 0.08	0.97
	Visit 3	0.01	− 0.06 to 0.08	0.92
	Visit 4	− 0.02	− 0.12 to 0.07	0.86
LA 30 Hz Flicker **PEAK**	Intercept	27.16	22.67 to 31.66	NA
	Visit 2	0.63	− 0.02 to 1.29	0.20
	Visit 3	− 0.03	− 0.88 to 0.82	0.97
	Visit 4	− 0.65	− 1.52 to 0.22	0.33
LA 3.0 ('Cone') a-wave **PEAK**	Intercept	13.83	13 to 14.67	NA
	Visit 2	0.33	0.16 to 0.49	**0.001**
	Visit 3	0.17	− 0.06 to 0.39	0.33
	Visit 4	0.14	− 0.09 to 0.37	0.50
LA 3.0 ('Cone') b-wave **PEAK**	Intercept	31.04	26.72 to 35.35	NA
	Visit 2	1.04	0.34 to 1.75	**0.025**
	Visit 3	− 0.02	− 1 to 0.96	0.97
	Visit 4	− 0.85	− 1.84 to 0.13	0.27
LA 3.0 b/a-wave **RATIO**	Intercept	2.64	1.58 to 3.7	NA
	Visit 2	− 0.20	− 0.46 to 0.06	0.33
	Visit 3	0.03	− 0.24 to 0.3	0.93
	Visit 4	0.34	0.02 to 0.66	0.14

The coefficients and 95% CIs for each visit quantify the change in that parameter relative to visit 1 (baseline). Corrected *p* values < 0.05, suggesting a significant difference to baseline, are highlighted in bold. CI, confidence intervals; DA, dark adapted; LA, light adapted.

Tables 3 and S4 show the results of GEE models analysing the effects of EDSS changes and clinical relapses in the preceding 12 months on ERG and OCT parameters, respectively. We observed a significant positive effect of EDSS changes on DA 3.0 b-wave peak times, with an increase in EDSS being associated with a prolongation of the ERG peak time of 3.65 ms (95%CI 1.78–5.53 ms). Throughout the study, eight patients experienced a significant change in EDSS at a total of 16 visits. All other effects were non-significant.

**Table 3:** Results of GEE models describing the effect of EDSS changes and recent (< 12 months) clinical relapses on ERG parameters over the study duration

ERG variable	Covariate	Coefficient	95% CI	P
DA 0.01 ('Rod') AMP	EDSS	12.17	− 33.64 to 57.97	0.77
	Relapse	17.19	− 15.72 to 50.11	0.61
DA 0.01 ('Rod') PEAK	EDSS	2.07	− 3.35 to 7.49	0.71
	Relapse	3.00	− 0.55 to 6.55	0.37
DA 3.0 ('Rod-Cone') a-wave PEAK	EDSS	0.10	− 0.22 to 0.41	0.76
	Relapse	0.02	− 0.25 to 0.29	0.89
DA 3.0 ('Rod-Cone') b-wave PEAK	EDSS	3.65	1.78 to 5.53	**0.004**
	Relapse	0.99	− 0.99 to 2.97	0.61
DA 3.0 b/a-wave RATIO	EDSS	− 0.20	− 0.42 to 0.03	0.37
	Relapse	0.09	− 0.02 to 0.19	0.37
LA 30 Hz Flicker PEAK	EDSS	1.05	− 0.54 to 2.63	0.50
	Relapse	0.14	− 1.04 to 1.32	0.89
LA 3.0 ('Cone') a-wave PEAK	EDSS	0.42	0.09 to 0.76	0.20
	Relapse	0.11	− 0.18 to 0.41	0.71
LA 3.0 ('Cone') b-wave PEAK	EDSS	0.86	− 0.7 to 2.41	0.60
	Relapse	− 0.25	− 1.58 to 1.08	0.83
LA 3.0 b/a-wave RATIO	EDSS	− 0.36	− 0.71 to -0.02	0.36
	Relapse	0.13	− 0.11 to 0.37	0.60

The coefficients and 95% CIs for each ERG variable quantify the effect of the covariates on the variable. Corrected *p* values < 0.05, suggesting a significant influence of the covariate on the relevant ERG parameter, are highlighted in bold. CI, confidence intervals; DA, dark adapted; EDSS, Expanded Disability Status Scale; LA, light adapted.

Finally, Tables S5 and S6 show the results of GEE models analysing the relationships between OCT parameters and their corresponding ERG parameters. All effects were non-significant.

## Discussion

Outer retinal function, as measured using the ERG, appears broadly stable in patients with MS and CIS over a three-year period at the group level. Although three ERG peak time parameters differed significantly relative to baseline (Table 2), the absolute differences were typically less than the temporal resolution of the ERG recording system (0.5 ms), with the smallest statistically significant change recorded being 0.33 ms. We, therefore, interpret these findings as representing fluctuation, rather than evidence of clinically meaningful changes to bipolar function. Supporting this interpretation, b-/a-wave amplitude ratios (a normalised measure of bipolar function) remained stable at the group level throughout the study (Fig. 2, Table S1). However, we cannot exclude the possibility that the heterogeneity of our patient cohort, reflective of the disease heterogeneity of MS generally, may have masked subtle changes to ERG parameters in different patient subpopulations. On a similar note, it is possible that differences in the disease course between patients may have contributed to our lack of significant findings (by different patterns of ERG findings in different patients being averaged out at the group level). This hypothesis is consistent with the positive correlation between changes in EDSS score and DA 3.0 (‘rod-cone’) b-wave peak time; despite the latter remaining stable in the cohort as a whole, it was prolonged in patients who experienced increased disability since their previous visit. It is also consistent with the variation shown by some individual patients, as seen in Fig. 2. However, recent non-ON relapses within the previous year were not associated with changes to ERG parameters. It remains possible that other clinical outcome measures (e.g. Multiple Sclerosis Functional Composite (MSFC) or 9-hole peg test) may better reflect longitudinal ERG findings in patients with MS.

In apparent contrast to our results, previous authors have documented a reduction in the amplitudes of several ERG responses, but stable ERG peak times, over a three-year period, with the greatest reduction (approximately 11%) being observed for the rod b-wave [10]. Sample size was comparable to the present work (52 vs. 45 MS eyes analysed); the patient cohort was also similar in terms of EDSS and disease duration, although ON eyes were excluded. Methodological differences (in particular, lack of information regarding the type and placement of ERG electrodes used, and fewer ERG examinations per patient [2 vs. 4 in the present work]) preclude a detailed comparison with the present work. An additional challenge in comparing the two studies is that we analysed only rod b-wave, rather than all standard ERG, amplitudes; however, our normalised measures of rod-cone and cone ERG amplitude (namely the b-/a-wave ratios) did not change significantly over the study period.

We recorded stable OCT parameters throughout the study at the group level. Whilst other authors have also recorded stable RNFL-G (but not RNFL-T or RNFL-PMB) over this period [23], thinning of this parameter seems to be more commonly reported [25, 27, 39]. The majority of previous longitudinal studies have not presented analyses of RNFL-T and RNFL-PMB [25, 27, 39]. Our lack of significant findings in this respect may be partially artefactual due to our modestly sized cohort, but likely also reflects the fact that only one of our patients experienced ON during the course of the study, as acute ON causes a reduction in RNFL thickness [40, 41]. As with RNFL, GCIP thickness has been found by other authors to decline longitudinally in patients with MS [23, 25, 27, 39], although not necessarily more so than in healthy control subjects [42]. Also as with RNFL, we interpret our lack of significant findings in this regard as a consequence of our sample size and the paucity of ON events over the course of the study. INL appears to remain stable longitudinally in eyes without recent ON [25, 27], as we observed here, but increases in thickness following ON [27], a finding which may also affect the contralateral (non-ON) eye [41]. These previous findings are compatible with our results describing stable INL, as only one of our patients experienced ON during the study period. We are unaware of previous longitudinal OCT studies of the retina distal to INL in patients with MS and CIS, and so our data represent the first evidence suggesting that OPL, ONL, and ORL may remain stable over three years in these patients. The lack of correlation between OCT results and corresponding ERG peak times also mirrors previous cross-sectional results [7]. Variability at the individual patient/eye level was observable for INL and OPL (Fig. 3); however, extrapolation of OCT findings at the group level to individual patients is considered problematic due to the resolution of commercial OCT devices (3–7 µm) being frequently greater than individual changes in retinal layer thicknesses [43]. With this in mind, we note that INL and OPL are the thinnest of the retinal layers studied here and thus may be the most challenging to reliably quantify longitudinally.

Our finding that increases in global disability (measured by EDSS) are associated with prolongation of DA 3.0 (‘rod-cone’) ERG b-wave peak times provides the first tentative evidence of a potential link between retinal bipolar cell function and MS disease activity. Despite this finding being driven in the present work by a relatively small number of patients (8 patients experiencing increases in EDSS at a total of 16 visits), we consider it biologically plausible due to the documented effects of autoimmunity on bipolar function [13–21]. Reproduction of this finding in a larger cohort would be desirable.

Our study has a number of limitations. Firstly, the modest size of our patient cohort is likely to have reduced the power of our analyses and may partially explain the predominance of non-significant findings in our results. Mitigating against this, our use of GEE enabled us to include both eyes of patients in our analyses, and thus to analyse a number of eyes comparable to that included in previous studies [8–10]. Given the length (approximately four hours per examination) and longitudinal nature of study visits, it was not feasible to recruit and examine a cohort of healthy control subjects alongside the patients. Additionally, we analysed functional measures only of the outer, rather than inner, retina, for several reasons. Firstly, inner retinal function in patients with MS has already been well described by other authors (e.g. [44–49]), as have VEP findings (e.g. [48–52]). Secondly, the outer retina contains the bipolar cells, whose cell bodies are found in the INL; functional changes to these cells are common in autoimmunity-mediated diseases other than MS, such as birdshot chorioretinopathy [13–17] and autoimmune retinopathy [18–21]. We were, therefore, primarily interested in the potential utility of the ERG as a longitudinal measure of bipolar function (assessed using the ERG b-wave) in MS. An additional consideration is that as the statistical power of our analyses was likely reduced due to the modest number of patients recruited, analysing additional parameters and further correction of multiplicity may have reduced the power of our analyses further.

Our analyses were primarily focussed on ERG peak times. This decision was made based on previous work by our group [7] and others [8–12, 22], in which peak times, and not amplitudes, were typically recorded as abnormal in patients with MS. However, we included rod ERG amplitudes in our analysis due to previous work suggesting longitudinal worsening of this parameter [10] (a finding we were unable to replicate, as discussed above). Additionally, amplitudes appear to be affected by placement of the DTL electrode, whereas peak times are not [32, 33]; subtle inter-visit differences in DTL position are, therefore, less likely to affect our results. Nevertheless, amplitude data were analysed in the form of the dark- and light-adapted 3.0 (rod-cone and cone, respectively) b-/a-wave ratios, which have the advantage of being normalised and thus independent of variations in DTL position; these ratios did not change significantly over the study duration.

In conclusion, despite a degree of individual variability, outer retinal function appears to remain approximately stable at the group level over a three-year period in patients with early or relatively benign MS and CIS. Increased global disability may be associated with bipolar cell dysfunction, although recent non-ON clinical relapses appear unrelated to retinal function. Future investigations may require a large patient cohort and/or an extended study duration in order to confirm potentially subtle functional changes.

## Supplementary Information

Below is the link to the electronic supplementary material.

Supplementary file1 (PDF 128 kb)

## Data Availability

Pseudonymised data may be provided upon reasonable request by qualified persons in writing to the corresponding author.
