# Supplementary material for: A three-year longitudinal study of retinal function and structure in patients with multiple sclerosis
Source: Doc Ophthalmol. 2021 Oct 27;144(1):3–16. doi: 10.1007/s10633-021-09855-7 (PMC8882570; doi:10.1007/s10633-021-09855-7)
Supplement: Supplementary file 1 — Supplementary file1 (PDF 128 kb) [file 10633_2021_9855_MOESM1_ESM.pdf]

SUPPLEMENTARY INFORMATION

James V. M. Hanson, Mei-Yee Ng, Helen K. Hayward-Koennecke, Sven Schippling, Kelly A. Reeve, Christina Gerth-Kahlert

A three-year longitudinal study of retinal function and structure in patients with multiple sclerosis

Corresponding author: James V. M. Hanson PhD, Department of Ophthalmology, University Hospital Zurich, Frauenklinikstrasse 24, 8091 Zurich, Switzerland. Email: james.hanson@usz.ch

Variable	Visit 1		Visit 2		Visit 3		Visit 4	
	Mean (SD)	Median [IQR]	Mean (SD)	Median [IQR]	Mean (SD)	Median [IQR]	Mean (SD)	Median [IQR]
n (eyes)	45	45	39	39	35	35	45	45
DA 0.01 ('Rod') **AMP**	392.36 (69.15)	402.10 [262.30, 544.90]	381.87 (50.58)	380.15 [266.80, 498.80]	361.78 (62.00)	345.20 [266.20, 498.00]	385.79 (62.99)	381.30 [266.60, 529.30]
DA 0.01 ('Rod') **PEAK**	79.69 (7.32)	78.50 [68.00, 99.50]	77.75 (7.00)	75.75 [67.00, 93.50]	79.94 (8.01)	79.50 [65.50, 98.50]	80.79 (6.03)	80.50 [68.00, 91.00]
DA 3.0 (Rod-Cone') a-wave **PEAK**	15.15 (0.72)	15.00 [13.50, 17.00]	15.58 (0.61)	15.50 [15.00, 17.00]	15.61 (0.64)	15.50 [14.50, 17.00]	15.69 (0.54)	15.50 [15.00, 17.00]
DA 3.0 ('Rod-Cone') b-wave **PEAK**	54.67 (3.42)	54.00 [48.00, 62.50]	54.82 (2.76)	53.75 [51.50, 62.50]	54.30 (3.52)	53.50 [48.50, 62.50]	55.10 (3.64)	54.50 [49.00, 62.50]
DA 3.0 b-/a-wave **RATIO**	1.62 (0.25)	1.53 [1.34, 2.57]	1.65 (0.23)	1.57 [1.35, 2.39]	1.71 (0.43)	1.60 [1.34, 3.31]	1.66 (0.24)	1.58 [1.37, 2.43]
LA 30Hz Flicker **PEAK**	28.63 (1.98)	28.50 [24.50, 34.00]	29.33 (1.86)	29.25 [26.50, 33.00]	28.71 (2.13)	28.50 [25.00, 33.00]	28.10 (2.10)	27.00 [24.50, 32.50]
LA 3.0 ('Cone') a-wave **PEAK**	14.37 (0.63)	14.50 [13.00, 15.50]	14.75 (0.48)	14.50 [14.00, 16.00]	14.61 (0.69)	14.50 [13.00, 16.00]	14.58 (0.54)	14.50 [13.50, 16.00]
LA 3.0 ('Cone') b-wave **PEAK**	31.77 (1.96)	32.00 [27.50, 36.00]	32.87 (1.74)	32.75 [30.00, 37.00]	31.84 (2.25)	32.00 [27.50, 37.00]	31.00 (1.90)	31.00 [27.50, 36.00]
LA 3.0 b-/a-wave **RATIO**	3.55 (0.62)	3.42 [2.54, 4.94]	3.37 (0.62)	3.44 [2.09, 4.66]	3.65 (0.48)	3.65 [2.46, 4.47]	3.95 (0.75)	3.94 [2.46, 6.61]

Table S1. Mean and median ERG results at Visits 1 (baseline), 2, 3, and 4. Amplitudes (‘AMP’) are given in millivolts, peak times (‘PEAK’) are given in milliseconds, and b-a-wave ratios (‘RATIO’) are unitless ratios of the amplitudes of the ERG b- and a-waves. DA, dark adapted; IQR, interquartile range; LA, light adapted; SD, standard deviation.

Variable	Visit 1		Visit 2		Visit 3		Visit 4	
	Mean (SD)	Median [IQR]	Mean (SD)	Median [IQR]	Mean (SD)	Median [IQR]	Mean (SD)	Median [IQR]
n (eyes)	43	43	38	38	35	35	45	45
RNFL-G	95.74 (16.15)	99.00 [38.00, 121.00]	96.89 (15.59)	99.50 [54.00, 122.00]	91.43 (17.46)	94.00 [35.00, 120.00]	92.82 (17.25)	95.00 [34.00, 119.00]
RNFL-T	63.58 (17.39)	63.00 [15.00, 101.00]	65.34 (16.11)	65.50 [30.00, 98.00]	61.71 (18.51)	62.00 [12.00, 98.00]	61.93 (16.74)	62.00 [17.00, 100.00]
RNFL-PMB	48.37 (13.01)	48.00 [6.00, 74.00]	50.18 (12.03)	52.00 [26.00, 73.00]	47.40 (14.31)	49.00 [4.00, 74.00]	47.49 (12.65)	48.00 [14.00, 77.00]
GCIPL	0.76 (0.13)	0.79 [0.35, 0.96]	0.78 (0.12)	0.80 [0.44, 0.93]	0.75 (0.15)	0.79 [0.33, 0.94]	0.75 (0.13)	0.77 [0.37, 0.95]
INL	0.38 (0.04)	0.37 [0.30, 0.44]	0.39 (0.03)	0.40 [0.32, 0.46]	0.39 (0.03)	0.39 [0.34, 0.45]	0.39 (0.03)	0.39 [0.33, 0.45]
OPL	0.27 (0.03)	0.27 [0.23, 0.38]	0.26 (0.04)	0.26 [0.21, 0.39]	0.26 (0.04)	0.25 [0.21, 0.37]	0.26 (0.03)	0.27 [0.21, 0.37]
ONL	0.68 (0.08)	0.69 [0.48, 0.80]	0.69 (0.09)	0.69 [0.48, 0.84]	0.68 (0.09)	0.68 [0.51, 0.84]	0.67 (0.09)	0.68 [0.49, 0.86]
ORL	0.77 (0.02)	0.77 [0.72, 0.81]	0.76 (0.02)	0.76 [0.72, 0.80]	0.77 (0.02)	0.77 [0.74, 0.82]	0.76 (0.02)	0.77 [0.71, 0.81]

Table S2. Mean and median OCT results at Visits 1 (baseline), 2, 3, and 4. RNFL-G, RNFL-T, and RNFL-PMB are thicknesses given in microns (μm); GCIPL, INL, OPL, ONL, and ORL are volumes given in cubic millimetres (mm^3) over a 3.45mm diameter circle. G, global average of RNFL thickness; GCIPL, ganglion cell-inner plexiform layer; INL, inner nuclear layer; ONL, outer nuclear layer; OPL, outer plexiform layer; ORL, outer retinal layers; PMB, papillomacular bundle; RNFL, retinal nerve fibre layer; T, temporal quadrant of RNFL.

OCT Variable	Covariate	Coefficient	95% CI	P
RNFL-G	Intercept	98.95	80.44 to 117.46	NA
	Visit 2	2.14	-2.74 to 7.03	0.67
	Visit 3	-2.18	-6.8 to 2.45	0.67
	Visit 4	-1.87	-5.73 to 1.99	0.67
RNFL-T	Intercept	65.64	37.83 to 93.46	NA
	Visit 2	1.77	-2.12 to 5.67	0.67
	Visit 3	-1.03	-4.89 to 2.84	0.80
	Visit 4	-1.61	-5.59 to 2.36	0.68
RNFL-PMB	Intercept	50.22	29.02 to 71.41	NA
	Visit 2	1.80	-1.3 to 4.9	0.67
	Visit 3	-0.40	-3.52 to 2.72	0.96
	Visit 4	-0.86	-3.95 to 2.23	0.80
GCIPL	Intercept	0.78	0.63 to 0.93	NA
	Visit 2	0.01	-0.03 to 0.05	0.71
	Visit 3	0.00	-0.04 to 0.04	0.99
	Visit 4	-0.01	-0.04 to 0.03	0.88
INL	Intercept	0.34	0.29 to 0.38	NA
	Visit 2	0.02	0 to 0.03	0.18
	Visit 3	0.01	0 to 0.03	0.28
	Visit 4	0.01	0 to 0.02	0.35
OPL	Intercept	0.26	0.22 to 0.31	NA
	Visit 2	-0.01	-0.02 to 0	0.65
	Visit 3	-0.01	-0.02 to 0	0.66
	Visit 4	-0.01	-0.02 to 0	0.35
ONL	Intercept	0.67	0.55 to 0.78	NA
	Visit 2	0.01	-0.01 to 0.04	0.67
	Visit 3	0.01	-0.01 to 0.03	0.67
	Visit 4	0.00	-0.02 to 0.02	0.99
ORL	Intercept	0.77	0.74 to 0.8	NA
	Visit 2	0.00	-0.01 to 0.01	0.99
	Visit 3	0.00	0 to 0.01	0.67
	Visit 4	0.00	-0.01 to 0.01	0.99

Table S3. Results of GEE models describing changes in OCT parameters over the study duration. The coefficients and 95% CIs for each visit quantify the change in that parameter relative to visit 1 (baseline). All comparisons to baseline were non-significant. CI, confidence intervals; G, global average of RNFL thickness; GCIPL, ganglion cell-inner plexiform layer; INL, inner nuclear layer; ONL, outer nuclear layer; OPL, outer plexiform layer; ORL, outer retinal layers; PMB, papillomacular bundle; RNFL, retinal nerve fibre layer; T, temporal quadrant of RNFL.

OCT Variable	Covariate	Coefficient	95% CI	P
RNFL-G	EDSS	4.06	-6.18 to 14.3	0.87
	Relapse	0.34	-7.14 to 7.83	0.93
RNFL-T	EDSS	3.68	-9.21 to 16.58	0.87
	Relapse	2.51	-6.85 to 11.87	0.87
RNFL-PMB	EDSS	2.67	-7.36 to 12.69	0.87
	Relapse	3.59	-3.56 to 10.75	0.87
GCIPL	EDSS	0.03	-0.07 to 0.13	0.87
	Relapse	0.01	-0.05 to 0.07	0.88
INL	EDSS	0.02	0 to 0.04	0.42
	Relapse	0.00	-0.02 to 0.02	0.87
OPL	EDSS	0.00	-0.02 to 0.02	0.88
	Relapse	-0.01	-0.03 to 0	0.44
ONL	EDSS	0.02	-0.05 to 0.09	0.87
	Relapse	-0.04	-0.09 to 0	0.42
ORL	EDSS	0.00	-0.02 to 0.01	0.87
	Relapse	0.00	-0.01 to 0.01	0.88

Table S4. Results of GEE models describing the effect of EDSS changes and recent (<12 months) clinical relapses on OCT parameters over the study duration. The coefficients and 95% CIs for each OCT variable quantify the effect of the covariates on the variable. All effects were non-significant. CI, confidence intervals; EDSS, Expanded Disability Status Scale; G, global average of RNFL thickness; GCIPL, ganglion cell-inner plexiform layer; INL, inner nuclear layer; ONL, outer nuclear layer; OPL, outer plexiform layer; ORL, outer retinal layers; PMB, papillomacular bundle; RNFL, retinal nerve fibre layer; T, temporal quadrant of RNFL.

ERG Variable	Covariate	Coefficient	95% CI	P
DA 0.01 (‘Rod’) **PEAK**	INL	14.92	-38.03 to 67.87	0.77
DA 3.0 (‘Rod-Cone’) b-wave **PEAK**	INL	6.37	-20.19 to 32.94	0.78
DA 3.0 (‘Rod-Cone’) b/a-wave **RATIO**	INL	-1.93	-3.98 to 0.12	0.37
LA 30Hz Flicker **PEAK**	INL	6.38	-13.56 to 26.33	0.76
LA 3.0 (‘Cone’) b-wave **PEAK**	INL	13.15	-6.61 to 32.92	0.50
LA 3.0 (‘Cone’) b/a-wave **RATIO**	INL	-1.85	-6.15 to 2.44	0.70

Table S5. Results of GEE models describing the effect of changes in INL on corresponding ERG parameters over the study duration. All effects were non-significant. CI, confidence intervals; DA, dark adapted; INL, inner nuclear layer; LA, light adapted.

ERG Variable	Covariate	Coefficient	95% CI	P
DA 3.0 (‘Rod-Cone’) a-wave PEAK	ONL	-0.17	-1.95 to 1.62	0.89
	ORL	5.68	-0.55 to 11.9	0.37
LA 3.0 (‘Cone’) a-wave PEAK	ONL	-0.17	-1.98 to 1.64	0.89
	ORL	4.90	-2.48 to 12.28	0.50

Table S6. Results of GEE models describing the effects of ONL and ORL on corresponding ERG parameters over the study duration. All effects were non-significant. CI, confidence intervals; DA, dark adapted; LA, light adapted; ONL, outer nuclear layer; ORL, outer retinal layers.
